# One more role for the brassinosteroid regulators: BZR1 and BES1 inhibit stomatal development in Arabidopsis cotyledons

**DOI:** 10.1093/plphys/kiae092

**Published:** 2024-02-20

**Authors:** Yee-Shan Ku

**Affiliations:** Assistant Features Editor, Plant Physiology, American Society of Plant Biologists; School of Life Sciences and Centre for Soybean Research of the State Key Laboratory of Agrobiotechnology, The Chinese University of Hong Kong, Hong Kong SAR, China

Stomatal density affects growth by regulating gaseous exchange and affects defense by controlling pathogen entry. The development of guard cells is initiated from the differentiation of meristemoid mother cells to become guard mother cells (GMCs), which further divide to form a pair of guard cells surrounding the stoma. The cell differentiation and division processes are regulated by various hormones, including brassinosteroid (BR). BR promotes guard cell formation in the hypocotyl but represses it in cotyledons (Wei et al. 2021). The tissue-specific effects suggest the delicate signaling regulation, which has remained unclear. In this issue of *Plant Physiology*, Li et al. report the role of BR in stomatal development in cotyledons in the dark (Li et al. 2024).

The homologous transcription factors (TFs) BZR1 and BES1 are the master BR signaling components (Wang et al. 2002; Yin et al. 2002; Li et al. 2018). The TFs also play a role in stomatal development, and their gain-of-function mutations (*bzr1-1D* and *bes1-D* respectively) inhibited stomatal development in Arabidopsis cotyledons (Li et al. 2024). To search for the target genes of BZR1 and BES1, the authors selected candidate stomatal development genes with BR regulatory elements in their promoters. *bzr1-1D* and *bes1-D* had promoted expression of *MKK9* but repressed expression of *FAMA*. *MKK9* encodes a motigen-activated protein kinase, while *FAMA* encodes a bHLH TF (Ohashi-Ito and Bergmann 2006; Lampard et al. 2009). BR inhibits stomatal development by promoting the expression of *MKK9* while repressing that of *FAMA* (Ohashi-Ito and Bergmann 2006; Li et al. 2024), and such regulation is mediated by BZR1 and BES1 (Li et al. 2024).

Further experiments were done to confirm the direct transcriptional regulation of the TFs on the target genes. Electromobility shift assay showed the bindings of BZR1 and BES1 to the target gene promoters in vitro. The in vivo binding was confirmed by chromatin immunoprecipitation. Using luciferase assays in protoplasts, the authors showed that the overexpression of *BZR1* promoted the expression of *MKK9* and repressed that of *FAMA*. To mimic the effects of BZR1 and BES1 on *MKK9* and *FAMA* expression and the resulting stomatal development phenotype, Arabidopsis lines expressing constitutively active MKK9 or having mutated FAMA were tested. Both lines showed inhibited stomatal development in the cotyledons.

The authors also locate BZR1, BES1, MKK9, and FAMA in the BR-mediated stomatal development signaling pathway. In BR signaling, BZR1 and BES1 are the known substrates of BIN2, which inactivates the TFs by phosphorylation and is repressed by BR (He et al. 2002; Zhao et al. 2002). BIN2 is a positive regulator of stomatal development: its gain-of-function mutation overproduces guard cells in etiolated cotyledons (Li et al. 2024). However, this phenotype could be rescued in *bzr1-1D* and *bes1-D* (Li et al. 2024). The results indicate that BIN2 acts upstream of BZR1 and BES1, inactivating these TFs and promoting stomatal development. Moreover, under the expression of constitutively active MKK9, the inhibition of BR synthesis failed to promote stomatal development. Such phenomenon suggested that MKK9 is downstream from BR. Based on the results, the authors suggested an updated BR regulatory pathway of stomatal development in Arabidopsis cotyledons. In the presence of BR, BIN2 is repressed. The repressed BIN2 fails to inactivate BZR1 and BES1. The active BZR1 and BES1 then promote *MKK9* expression and repress *FAMA* expression to inhibit stomatal development (Fig.).

**Figure:**
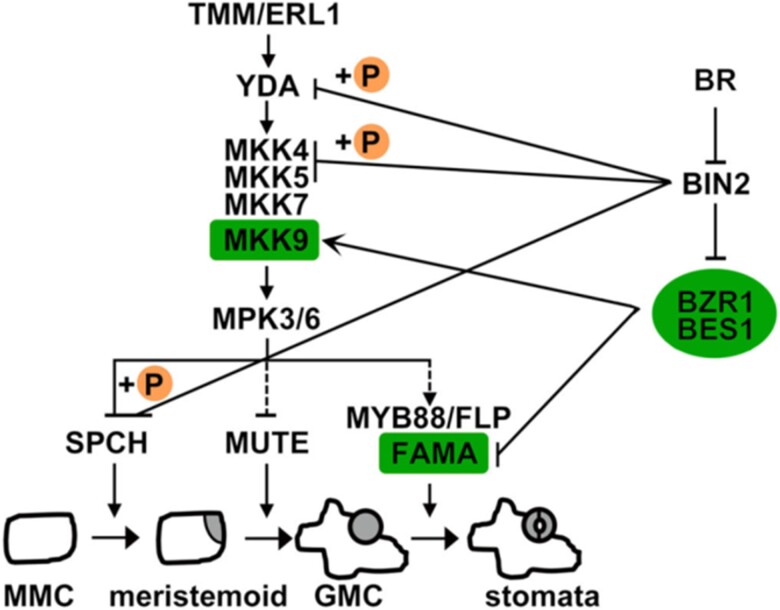
The updated BR signaling pathway for stomatal development regulation in Arabidopsis cotyledons. In the presence of BR, BIN2 is repressed. The repressed BIN2 fails to inactivate BZR1 and BES1. The active BZR1 and BES1 then promote *MKK9* expression and repress *FAMA* expression. The promoted *MKK9* expression may inhibit the transition from meristemoid to GMC. The repressed *FAMA* expression fails to promote the development from GMC to guard cells. This figure is adapted from Li et al. (2024).

BR has been known to regulate stomatal development via the action of BZR1 and BES1. In this study, the authors integrated BZR1 and BES1 into the BR-mediated stomatal development signaling pathway in Arabidopsis cotyledons, identified *MKK9* and *FAMA* as the bona fide targets of BZR1 and BES1, and updated the pathway with these components.

## Dive Curated Terms

The following phenotypic, genotypic, and functional terms are of significance to the work described in this paper:

BZR1 Gramene: AT1G75080
BZR1 Araport: AT1G75080

BES1 Gramene: AT1G19350
BES1 Araport: AT1G19350

BIN2 Gramene: AT4G18710
BIN2 Araport: AT4G18710

MKK9 Gramene: AT1G73500
MKK9 Araport: AT1G73500
